# Renal function during tenofovir‑based antiretroviral therapy among people living with HIV in Lilongwe, Malawi: findings from the prospective LighTen cohort study

**DOI:** 10.1186/s12879-026-12675-2

**Published:** 2026-01-28

**Authors:** Melani R. Mahanani, Florian Neuhann, Ethel Rambiki, Angelina Nhlema, Hannock Tweya, Myo Chit, Jane Chiwoko, Thom Chaweza, Claudia Wallrauch, Tom Heller, Volker Winkler, Gerd Fätkenheuer, Hans-Michael Steffen

**Affiliations:** 1 Heidelberg Institute of Global Health, Heidelberg University Hospital, 69120, Im Neuenheimer Feld 130.3, Heidelberg, Germany; 2 Centre for Preventive Medicine and Digital Health, Division of Prevention of Cardiovascular and Metabolic Diseases, Medical Faculty Mannheim, Heidelberg University, Mannheim, Germany; 3 School of Medicine and Clinical Sciences, Levy Mwanawasa Medical University, Lusaka, Zambia; 4 Lighthouse Clinic, Kamuzu Central Hospital, Lilongwe, Malawi; 5 International Training and Education Center for Health (I-TECH), Lilongwe, Malawi; 6 Institute of Infectious Diseases and Tropical Medicine, LMU University Hospital, LMU Munich, Germany; 7 International Training and Education Centre for Health, University of Washington, Seattle, WA USA; 8 Division of Infectious Diseases, Department I of Internal Medicine, Faculty of Medicine, University of Cologne, University Hospital Cologne, Cologne, Germany; 9 Clinic for Gastroenterology and Hepatology, Faculty of Medicine, University of Cologne, University Hospital Cologne, Cologne, Germany; 10 Hypertension Center, Faculty of Medicine, University of Cologne, University Hospital Cologne, Cologne, Germany; 11 Department of Postgraduate Studies and Research, Chreso University, Lusaka, Zambia

**Keywords:** HIV, Antiretroviral therapy, Renal function, Glomerular filtration rate

## Abstract

**Background:**

Most first-line antiretroviral therapy (ART) regimes in Sub-Saharan Africa contain tenofovir disoproxil fumarate (TDF) which has a nephrotoxic potential. Baseline renal function assessment is not feasible in many settings and therefore not required prior to starting ART according to Malawian guidelines. We assessed renal function over 36 months in people living with HIV (PLHIV) starting TDF-based ART at Lighthouse Clinic, Lilongwe, Malawi.

**Methods:**

Data on demographics, medical history, laboratory values, WHO stage, and anthropometric measures were collected at study entry and during visits at 1, 3, 6 months, and every 6 months until month 36. The main outcome of the study was renal function, defined by the estimated glomerular filtration rate (eGFR) calculated using the CKD-EPI equation. Descriptive statistics and multivariable linear regression analysis were performed.

**Results:**

A baseline creatinine value was available for 1,430 PLHIV (57% female, mean age ± SD 36.0 ± 9.3 years). Of these, 443 PLHIV (62% female) were observed until month 36. Factors associated with changes in eGFR over time included baseline log-transformed eGFR (coefficient = −0.787; *p* < 0.001), MAP at baseline, and initial WHO clinical HIV stage. The overall trend in eGFR categories indicated a shift towards lower classes.

**Conclusions:**

In this large cohort of PLHIV, TDF did not result in a decline in eGFR. Indeed, an unexpected trend of improved renal function was observed. Even though findings need to be interpreted with caution due to considerable attrition, our results suggest TDF can be administered safely in resource-limited settings where ART decisions are frequently made without pre-treatment renal assessment.

**Supplementary Information:**

The online version contains supplementary material available at 10.1186/s12879-026-12675-2.

## Introduction

The HIV/AIDS epidemic remains a significant public health challenge in sub-Saharan Africa (SSA), which continues to bear the highest burden of the disease globally. According to the World Health Organization (WHO) in 2022, approximately 25.6 million people were living with HIV (PLHIV) in SSA [1] and about 380,000 (60%) of all AIDS-related deaths had occurred here [2].

Antiretroviral therapy (ART) is a cornerstone in the fight against HIV/AIDS, transforming the disease from a fatal condition to a manageable chronic illness. The introduction and expanded access of ART have significantly reduced AIDS-related deaths, improved the quality of life for PLHIV [3], and played a critical role in preventing HIV transmission [4]. Despite these advancements, challenges remain, particularly in resource-limited settings where access to healthcare and diagnostics can be constrained.

Following the WHO recommendations on ART [5], countries in SSA adopted first-line ART regimens which typically include a backbone of two nucleoside reverse transcriptase inhibitors (NRTIs) in combination with an anchor drug, mostly non-nucleoside reverse transcriptase inhibitors (NNRTIs) or an integrase inhibitor (INSTI). Across the region tenofovir disoproxil fumarate (TDF), an NRTI, is a key component of most first-line ART regimens and in recent years also in second line ART [6]. However, there are concerns regarding its long-term safety profile, particularly its potential nephrotoxic effects. Evidence has indicated that TDF use is associated with a decline in estimated glomerular filtration rate (eGFR), a key indicator of renal function. A meta-analysis [7] reported varying degrees of decrease in eGFR among patients on TDF-based ART compared to those on other ART regimens and incident chronic kidney disease was found to be infrequent in those with low baseline risk [8, 9].

One study conducted in South Africa showed that TDF toxicity was related to pre-existing renal pathologies [10]. Besides non-communicable diseases (NCD) which contribute considerably to chronic kidney disease, HIV itself plays a critical role as risk factor [11]. HIV-related acute or chronic kidney diseases can arise either directly due to the viral infection in the kidney and/or indirectly through drug-related effects [12]. In general, evidence from SSA regarding chronic kidney disease prevalence is limited, with studies reporting rates ranging from 6% to 48.5% across different countries, making comparisons challenging [13].

Acknowledging the potential risks of nephrotoxicity, global and regional guidelines provide specific recommendations for the use of TDF in PLHIV. WHO and Malawi’s national guidelines [14] advise that TDF should be used with caution in individuals with pre-existing renal impairment or those at high risk for renal dysfunction. To assess renal function, various equations are available such as the Cockcroft-Gault equation (CG) to calculate creatinine clearance, and the Modification of Diet in Renal Disease equation (MDRD) or the Chronic Kidney Disease Epidemiology Collaboration equation (CKD-EPI) to quantify eGFR [15]. Despite the availability of specific guidelines and various equations to monitor kidney function, the implementation of these measures is often impractical in low-income countries mainly due to unavailability of creatinine values.

Malawi has one of the highest HIV burdens in SSA, with a prevalence among adults (≥ 15 years) at 8.9% corresponding to approximately one million PLHIV [16]. The national HIV program has made significant strides in the fight against HIV and is one of the first countries to have adopted ART regimens that contain TDF as first line ART in 2019. TDF-based ART regimens have formed the backbone of the program’s first line ART since 2011 [17]. Recognising the limitations with access to laboratory services in the primary health care facilities, Malawi adopted the public health approach where all patients with a new HIV positive diagnosis can be initiated on ART in the absence of routine baseline laboratory tests. However, the program adopted annual viral load screening among those on treatment for monitoring [18]. Other laboratory tests are available in response to the patient’s presentation whilst on treatment and are often managed at secondary and tertiary levels of health care where laboratory services are available. This limitation poses challenges for healthcare providers and policymakers striving to optimize ART regimens while ensuring patient safety.

Here we present an analysis from the Lighthouse Tenofovir cohort study (LighTen study), a prospective observational study at Lighthouse Clinic, that aimed to assess baseline renal function and the long-term impact of TDF on kidney function in an HIV-infected, treatment-naïve patient cohort initiating TDF-based ART.

## Methods

The LighTen study (Clinical Trial number NCT02381275, registration date 2015-03-06) included adult PLHIV presenting for ART initiation to the Lighthouse Clinic, Lilongwe, Malawi. Recruitment started in August 2014 and follow-up ended in October 2019. The study was approved by the Ethical Committees of the involved German universities of Cologne (No. 14–251) and Heidelberg (No. S-293/2014) and the National Health Research Commission of the Ministry of Health, Malawi (Appr. No. NHSRC Protocol #1199).

### Setting

The Lighthouse clinic is situated at Kamuzu Central Hospital (KCH), the major referral hospital serving the central region in Malawi. It is a public clinic without treatment fees and follows national guidelines in clinical management of HIV. As per Malawi HIV treatment guidelines at the time of enrolment [19], all patients newly initiating ART received a fixed-dose combination of 300 mg TDF, 300 mg lamivudine (3TC) and 600 mg efavirenz (EFV). At Lighthouse, most routine clinical care is provided by nurses and clinical officers who complete three to four years of basic training. The Lighthouse clinic uses an electronic medical records system, where routine clinic visits data are captured in real-time while laboratory data are entered retrospectively.

### Study population

Adult patients (≥ 18 years) with confirmed HIV infection and no self-reported history of prior ART, willing to participate and able to give written informed consent were included. Participant demographics, medical history including self-reported hypertension, smoking status, laboratory data, current medication, and anthropometric data (height, weight) were collected at study entry as well as at regular visits at one, three, six months and every six months thereafter until month 36. Weight was measured with shoes removed and only light clothing.

Office blood pressure (BP) was determined during each visit as the mean of the following measurements: (1) oscillometric BP measurement (Rossmax CF115f and Omron M300) as part of anthropometric measurements after entering the clinic, and (2) additional measurement by a qualified nurse if the initial BP was ≥ 140/90 mmHg. All measurements were done in the sitting position 5 min after placement of the cuff to the dominant upper arm [20]. The BP was classified according to the 2018 European Society of Cardiology/European Society of Hypertension treatment guidelines [20]. Participants diagnosed with hypertension were further treated according to the Malawi Standard Treatment Guideline [19].

Participants who stopped or changed ART regimen for any reason were censored at the date of treatment change as were participants who withdraw their consent, were transferred to another HIV treatment centre or had missed two or more appointments. Following national guidance, all participants received cotrimoxazole prophylaxis while isoniazid preventive therapy was given only to a minority of participants.

### Laboratory analysis

All participants had baseline laboratory evaluations beyond the standards of the Malawian HIV treatment program, including full blood count (AcT 5diff CP Hematology Analyzer, Beckman Coulter, Atlanta GA, USA), liver and renal function tests (Erba XL200, Erba Mannheim, Germany [Kamuzu Central Hospital laboratory], Mindray Bs 120 Chemistry Analyser, China [Lighthouse laboratory], and Siemens ADVIA Chemistry XPT System [Heidelberg laboratory] using standard operating procedures according to the manufacturers’ instructions), CD4 cell counts (Pima CD4-test, Abbott, Cape Town, South Africa), and HIV-RNA plasma levels (Cepheid GeneXpert, HIV viral load, Sunnyvale, CA, USA). CD4 counts and viral load were measured every 6 and 12 months, respectively. Proteinuria was measured using Siemens urine dipstick tests (Siemens, Germany) on freshly collected urine. As the procurement of laboratory reagents was interrupted, serum samples stored at -80 °C had to be sent in different shipments to an outside laboratory for further analyses. However, this process was interrupted during the Covid-19 epidemic. To ensure comparability, creatinine data were adjusted to the upper limit of normal of the respective laboratory.

### Study outcome and definition

The main outcome of the study was the change in renal function which was assessed by the eGFR calculated using the CKD-EPI equation without correction factor for people of African origin [21] as at the inception of the LighTen study this formula had been shown to provide the most accurate estimation [21–23].

### Statistical analysis

Descriptive statistics were calculated to understand the characteristics of the study cohort at study entry for the whole study population and for the subgroup observed until month 36. The statistics included the mean and standard deviation (for normally distributed variables), median and interquartile range (for non-normally distributed variables), and frequencies and percentages for categorical variables.

Data on sex (binary; 0: female, 1: male), age at baseline (in years; continuous), months of visit after study enrolment (0 to 36; continuous), body weight and height (continuous), BP (systolic and diastolic; continuous), renal function parameter (eGFR according to the CKD-EPI equation, continuous), WHO HIV stage (categorical; 1, 2, 3, 4), proteinuria from urine dipstick tests (binary; 0 or 1+), CD4 count (continuous), viral load (continuous) were available for inductive analyses. Mean arterial pressure (MAP) was calculated ([2xdiastolic BP + systolic BP]/3).

We further examined the trend of eGFR over time using scatter plot and linear regression. Due to the non-normal distribution of eGFR at baseline, median and interquartile range were used for descriptive statistics. For longitudinal analysis, mean eGFR was plotted over time to evaluate trends from month 0 to 36, providing a consistent summary measure for population-level changes despite individual skewness.

Following this, we assessed factors associated with changes in eGFR from month 0 to month 36 in five different multivariable linear regression models. The baseline model (Model 1) included only log(eGFR) and MAP, both at baseline. For each subsequent model, we added one additional variable—WHO HIV stage (Model 2), dipstick proteinuria (Model 3), baseline CD4 count (Model 4), and log(viral load) at baseline (Model 5)—incrementally. However, while this approach allowed us to assess the individual effect of each variable as it was introduced the number of cases dropped considerably due to missing values.

Additionally, eGFR classifications were compared between study entry and final visit and illustrated using a Sankey diagram in 12 months intervals.

For the sensitivity analysis, adjusted creatinine was selected as outcome as it was independent of the measurement source and less influenced by the assumptions inherent in eGFR calculations and hence allowed to assess the robustness of our findings; results of the sensitivity analysis are presented in the appendix.

All statistical analyses used a 0.05 significance level. All data analyses were performed using Stata/IC 15.1 (StataCorp LLC, 4905 Lakeway Drive, College Station, TX 77845, USA).

## Results

A total of 1,433 participants were enrolled during the two-year recruitment period, of whom a baseline creatinine value was available for 1,430 PLHIV (814 females, 56.9%). 832 PLHIV could be observed until month 36 and out of these creatinine data were documented in 443 cases (274 females; 61.9%) at the final visit with viral replication below the limit of detection in 428 cases (96.6%). Reasons for drop-out included transfer out, study withdrawal, defaulting, change of ART and deaths. Baseline characteristics of the study population are given in Table 1. When comparing enrolled versus not enrolled patients during the recruitment period those who took part in the LighTen study were older with more overweight and advanced cases as well as better survival and monitoring during follow-up (see Suppl. Table A1).

**Table 1:** Characteristics at study entry for the whole study population and the subgroup observed until month 36

Characteristics	All observations		Visited month 36	
	*n*	%	*n*	%
Total	1430	100.0	443	100.0
Sex				
Female	814	56.9	274	61.9
Male	616	43.1	169	38.2
Age [years] (mean; SD^a^)	36.0; 9.3		37.8; 9.1	
Age group [years]				
18–24	134	9.4	20	4.5
25–34	561	39.2	157	35.4
35–44	490	34.2	165	37.3
45–54	186	13.0	83	18.7
55–64	48	3.4	14	3.2
65+	11	0.8	4	0.9
^c^eGFR [mL/min/1.73m^2^] (median; IQR^b^)	102; 83–116		101; 83–114	
eGFR category (eGFR [mL/min/1.73m^2^])				
G1 (≥ 90)	964	67.4	291	65.7
G2 (60–89)	387	27.1	136	30.7
≥G3 (< 60)	79	5.5	16	3.6
Adjusted creatinine (median; IQR)	0.758; 0.644–0.873		0.745; 0.638–0.869	
Dipstick proteinuria ^d^				
0	1092	76.4	361	81.5
1 + ^d^	247	17.3	62	14.0
Missing	91	6.3	20	4.5
Systolic blood pressure [mmHg] (mean; SD)	117.2; 20.1		120.0; 19.5	
Diastolic blood pressure [mmHg] (mean; SD)	75.1; 13.7		76.6; 13.1	
Mean arterial pressure [mmHg] (mean; SD)	89.1; 15.1		91.1; 14.4	
Blood pressure categories (ESH Classification)				
Optimal	746	52.2	208	46.9
Normal	278	19.4	100	22.6
High-normal	170	11.9	45	10.2
Hypertension stage 1	163	11.4	67	15.1
Hypertension stage 2	30	2.1	9	2.0
Hypertension stage 3	34	2.4	14	3.2
Missing	9	0.6	0	0.0
Weight [kg] (mean; SD)	59.9; 11.9		60.0; 11.2	
Body mass index categories				
Critically low	81	5.7	19	4.3
Normal weight	862	60.3	264	59.6
Overweight	318	22.2	107	24.1
Obese	164	11.5	51	11.5
Missing	5	0.3	2	0.5
WHO HIV stage				
1 or 2	880	61.5	335	75.6
3 or 4	550	38.5	108	24.4
CD4 count [cells/mm^3^] (median; IQR)	268; 114–425		272; 134–412	
Viral load [copies/mL] (median; IQR)	38,201; 6919–181,280		39,150; 9526–148,281	

^a^*SD* standard deviation; ^b^*IQR* interquartile range; ^c^*eGFR* estimated glomerular filtration rate; ^d^Dipstick proteinuria (1 + or more)

**Fig. 1:**
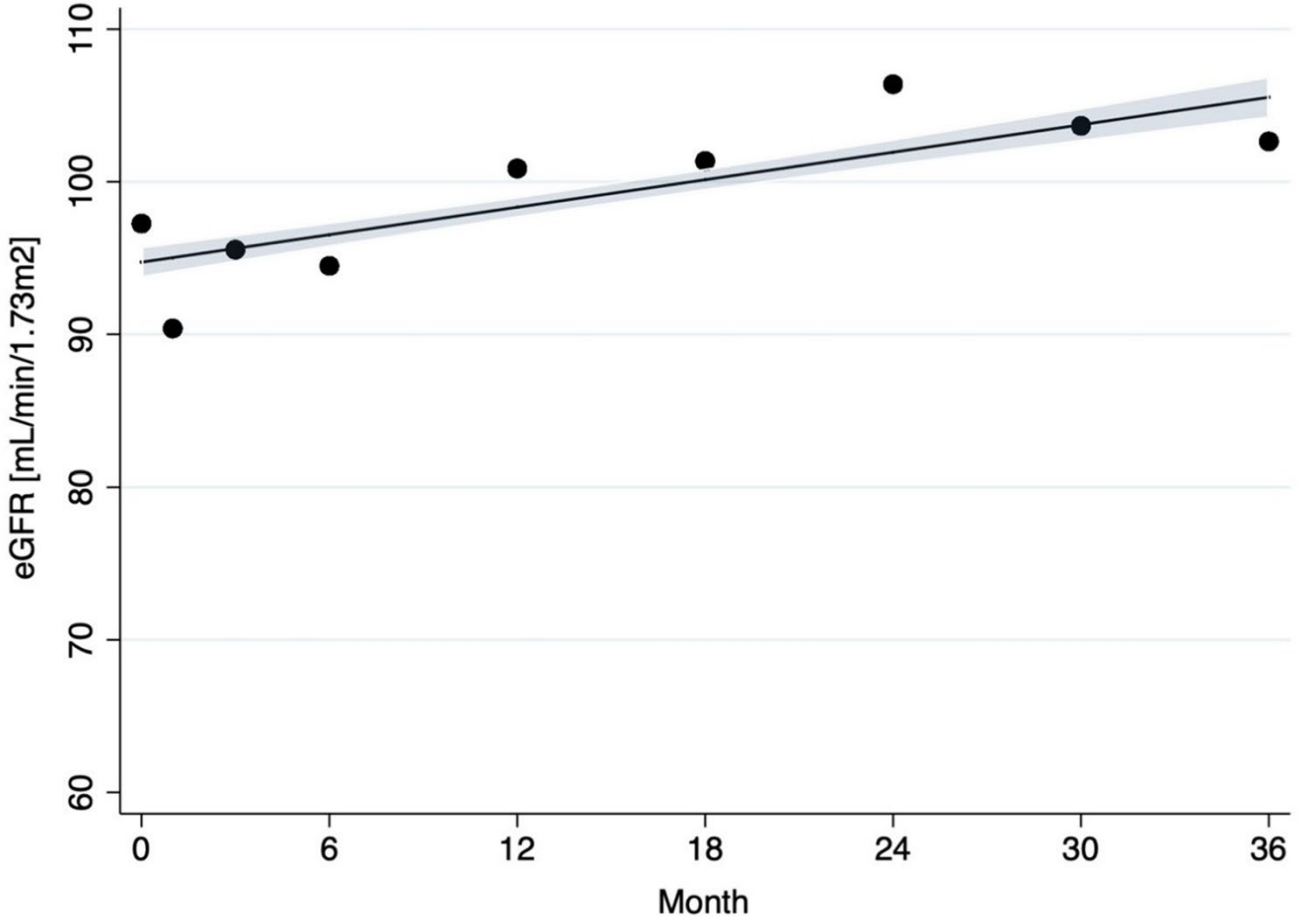
Mean eGFR over time from month 0 to month 36, with modelled line and 95% confidence interval among PLHIV observed for 36 months. The number of individuals with documented creatinine results at each time point were: baseline (*n* = 1430), month 1 (*n* = 980), month 3 (*n* = 877), month 6 (*n* = 893), month 12 (*n* = 704), month 18 (*n* = 550), month 24 (*n* = 518), month 30 (*n* = 417), and month 36 (*n* = 443)

**Fig. 2:**
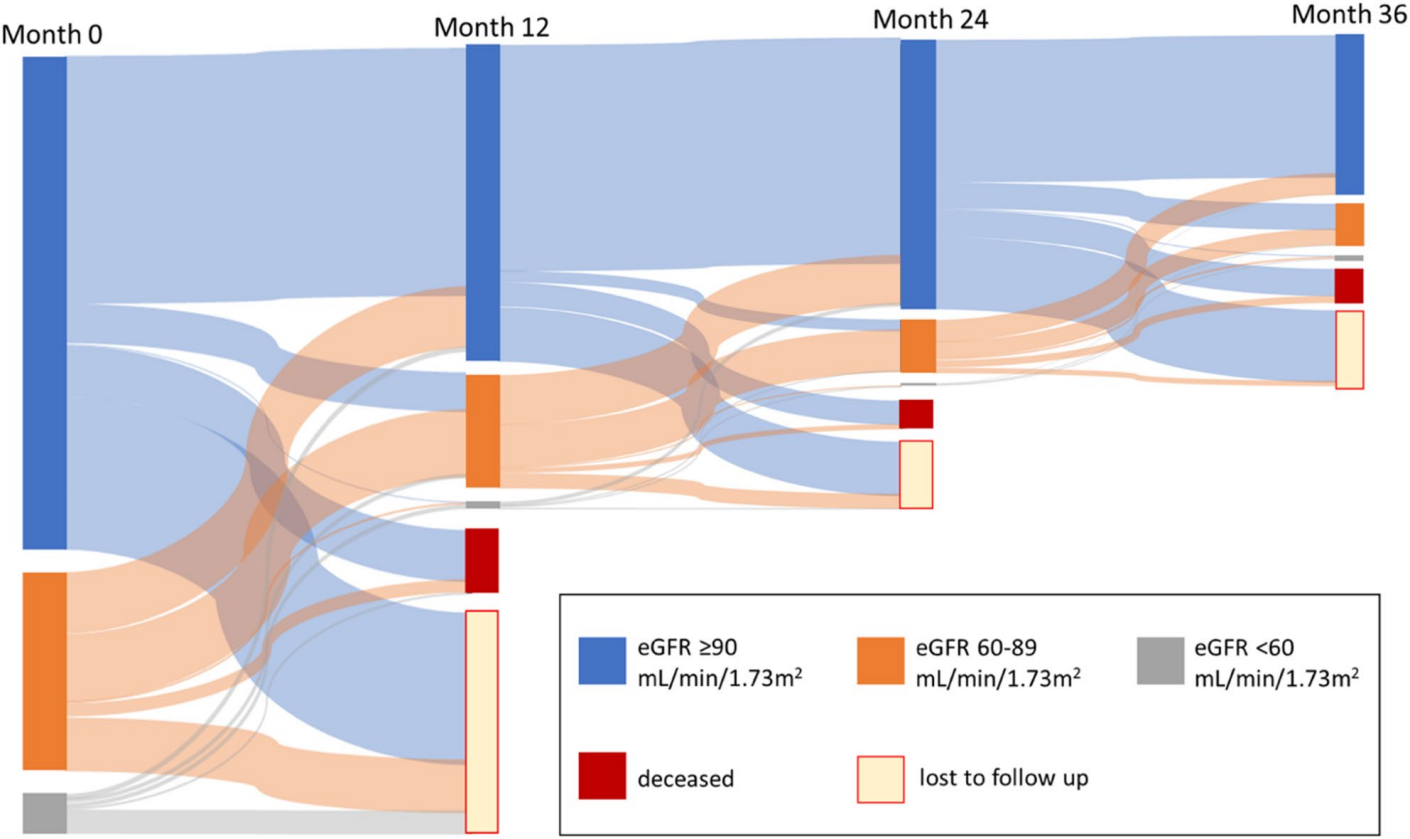
Development of eGFR classes over time

Following the initiation of TDF-based ART a gradual increase in mean eGFR until the end of the observation period could be observed (see Fig. 1) and the change in eGFR categories over time showed a clear trend towards lower classes, i.e., improved renal function (Table 2; Fig. 2). There was no discernible sex-related difference in the change of eGFR over time (see Suppl. Figure A1). Table 3 presents the results of the multivariable analyses. log(eGFR) and MAP at baseline (for all models) and the initial WHO clinical HIV stage (for Models 2, 3, and 4) were factors associated with eGFR changes over time. No significant effect was observed for proteinuria measured using urine dipstick tests, CD4 count or viral load at baseline. Also, when adjusted serum creatinine was analysed, again a trend towards lower values (indicating improved renal function) at 36 months was observed (see suppl. figure A2) and multivariable regression analyses identified the same associated factors except for WHO clinical stage at entry (see Suppl. Table A2).

**Table 2:** eGFR class at study entry and the final visit for PLHIV observed for 36 months

eGFR [mL/min/1.73m^2^]	Study entry	Final visit
≥ 90	65.7%	77.4%
60–89	30.7%	20.8%
30–59	2.5%	1.8%
15–29	0.9%	0.0%
< 15	0.2%	0.0%

**Table 3:** Multivariable linear regression for changes in eGFR over time from month 0 to month 36

Variables		Model 1 *n* = 443		Model 2 *n* = 443		Model 3 *n* = 368		Model 4 *n* = 368		Model 5 *n* = 352	
		ß	*p*-value	ß	*p*-value	ß	*p*-value	ß	*p*-value	ß	*p*-value
log(eGFR^a^) at baseline		-0.779	< 0.001	-0.783	< 0.001	-0.788	< 0.001	-0.789	< 0.001	-0.787	< 0.001
MAP at baseline		-0.002	0.002	-0.002	0.001	-0.002	0.008	-0.002	0.008	-0.002	0.002
WHO HIV stage	1 or 2	-	-	Ref.	0.016	Ref.	0.038	Ref.	0.034	Ref.	0.060
	3 or 4	-	-	-0.048		-0.048		-0.050		-0.042	
Dipstick proteinuria	0	-	-	-	-	Ref.	0.781	Ref.	0.757	Ref.	0.632
	1 + ^b^	-	-	-	-	-0.013		-0.014		-0.022	
CD4 count at baseline	≥ 200	-	-	-	-	-	-	Ref.	0.456	Ref.	0.371
	< 200	-	-	-	-	-	-	0.044		0.053	
log(viral load) at baseline	< 4	-	-	-	-	-	-	-	-	Ref.	0.089
	4 to < 5	-	-	-	-	-	-	-	-	-0.157	
	5 to < 6	-	-	-	-	-	-	-	-	0.064	
	≥ 6	-	-	-	-	-	-	-	-	0.115	
Constant		3.782	< 0.001	3.820	< 0.001	3.827	< 0.001	3.832	< 0.001	3.738	< 0.001

^a^*eGFR* estimated glomerular filtration rate; ^b^Dipstick proteinuria (1 + or more)

At study entry 57 PLHIV had an eGFR < 50 ml/min and thus an indication to dose adjustment or change to a different antiretroviral treatment regimen, however, only eight patients were switched away from TDF. Since 24 patients were transferred out, withdrew their consent or defaulted, definite outcomes could be ascertained for 33 of these 57 PLHIV: 4 out of 8 patients who according to guidelines switched-out TDF at month 1 (2 deaths within 14 days and 2.5 months, respectively; 2 survivors) and in 29 out of 49 patients who yet continued TDF (7 deaths within 10 days to 7 months, the latter despite normalized eGFR; 22 survivors).

## Discussion

The present study demonstrated that TDF did not result in a discernible decline in renal function of 443 PLHIV observed over a period of 36 months. Additionally, it provided empirical evidence from real life experience in a resource-limited setting in Malawi, confirming a low prevalence (5.5%) of moderate to severe renal dysfunction, i.e. eGFR < 60 ml/min [24, 25]. Our observations indicate that several baseline factors were significantly associated with changes in eGFR over time. Specifically, baseline eGFR and MAP were negatively associated with subsequent eGFR changes. Thus, individuals with higher initial eGFR values or higher MAP as surrogate marker for arterial hypertension with its known unfavourable effects on the kidneys, experienced larger declines in renal function as did participants classified at WHO HIV stages 3 or 4, compared to those in earlier disease stages. Despite these associations, the overall trend in eGFR categories indicated a shift towards lower classes, reflecting an improvement in renal function across the study population. These findings provide reassurance for the use of TDF-based ART in resource limited settings, where ART decisions often need to be made without pre-treatment renal function assessments.

Our findings diverge from a substantial body of literature that has documented potential nephrotoxic effects in TDF-based ART. A recent systematic review [7] has reported an association between TDF-based ART use and increased risk of renal impairment from a total of 17,282 PLHIV across ten studies in nine countries. It was concluded that long-term TDF-based ART can lead to tubular disease and chronic kidney disease (CKD), although in three studies TDF did not increase the odds of CKD. Other markers of renal impairment have also been reported to be associated with TDF in other studies, such as tubular proteinuria [26] and proximal tubular dysfunction [27].

Debeb et al. [28] have assessed TDF-associated renal dysfunction among PLHIV in Ethiopia, which shares similar sociodemographic characteristics to Malawi. Baseline characteristics were observed to be associated with decreased renal function, including being older than 55 years, concurrent nephrotoxic drug use, and combined use of ritonavir-boosted protease inhibitors. This finding aligns with other studies conducted in populations with higher baseline risks for renal disease [29–31], which may not fully represent the diverse conditions in real-world, resource-limited environments.

Mtisi et al. [32] have also extensively summarised studies on TDF-associated kidney diseases among PLHIV, focusing on various African populations. Analysing 31 studies from 14 countries, this systematic review highlighted conflicting evidence of the association of TDF with renal dysfunction, with half suggesting the overall safety of TDF, while the other half reported varying degrees of renal toxicity. Furthermore, there was no uniformity in outcome measures, and many studies did not specifically test for proximal tubular dysfunction. Notably, Malawi was not included in this systematic review, underscoring the need for studies from this population to contribute to the evidence base.

In our study, renal function improved or remained stable for almost all patients with reduced baseline eGFR, again confirming similar findings from earlier studies in Malawi [25] as well as Namibia [31], Zambia [23, 33], or Brazil with long-term follow-up [34]. It is probable that the initiation of ART, including TDF, contributed to overall health improvements of PLHIV, such as the reduction of HIV-associated inflammation [35], which could positively impact renal function. In addition, the benefits of viral suppression and immune recovery [36] could have outweighed any potential nephrotoxic effects of TDF. Although a systematic review [37] has explored evidence on the association of pro-inflammatory markers with kidney dysfunction in PLHIV on highly active ART, this study has also recognised the presence of conflicting evidence on the effects of ART, which may include TDF, on inflammation markers in PLHIV.

Another consideration is the potential role of patient selection and adherence. Although renal function was assessed at the study baseline, patients who remained in our study and adhered to their ART regimen might represent a relatively healthier subset of the overall population, which could partially explain the positive renal outcomes observed. A study among PLHIV in Ghana has shown an initial increase in eGFR over the first year of taking ART. Factors related to this finding might be due to many participants being acutely unwell at diagnosis, followed by significant improvement after the initiation of ART. Survivor bias may have also improved mean eGFR changes if PLHIV with poorer outcomes did not survive and their data were missed [38].

One of the primary strengths of this study lies in its practical applicability and relevance. In contexts where routine pre-treatment renal function testing is not feasible, our results suggest that TDF can be used safely without a high risk of renal impairment. This is especially relevant given the widespread use of TDF as a first-line agent in ART regimens across the region. Moreover, this study reflects the challenges and complexities of providing ART in resource-limited settings, including issues related to infrastructure, access to care, and patient follow-up. The findings suggest that, even without routine renal function monitoring, TDF can be used safely in these environments, which is crucial information for healthcare providers and policymakers.

One of the major limitations of our study is the considerable high attrition rates. Such occurrences have been observed to be particularly common in long-term cohort studies in resource-limited settings where participants often encounter barriers such as financial constraints, transportation difficulties, and competing health priorities. The loss of participants from the study could introduce bias, as those who remained in the study and adhered to their ART regimen might represent a relatively healthier subset of the overall population, which could partially explain the positive renal outcomes observed. Another limitation relates to the variability of the eGFR due to the measurements in different laboratories. However, when using creatinine values adjusted to the upper limit of normal of the individual laboratory our findings remained essentially unchanged, thus strengthening the credibility of our findings.

Given these limitations, it is crucial to interpret the findings with caution. While the data suggest that TDF is safe and may even improve renal function in this cohort, the potential for attrition bias means that these results may not be fully generalisable to all PLHIV in similar settings. PLHIV who visited month 36 were more often in WHO stages 1 or 2 and showed less proteinuria, factors known to be associated with less renal toxicity while on TDF [39, 40]. However, they were also older and more often female, factors associated with a higher risk for renal toxicity [9]. In addition, the study did not include a control group of PLHIV on non-TDF-based regimens, which limits our ability to directly attribute the observed renal outcomes to TDF alone. Other factors, such as improvements in overall healthcare access, management of comorbidities, and patient adherence to ART, could also have played a significant role.

Future research should aim to address the limitations identified in this study, particularly the issue of attrition. Moreover, further longitudinal studies should consider a range of factors, including the impact of co-infection, other medications, and demographic variables on renal outcomes. Given the potential for improved renal function observed in our study, it would be valuable to explore this phenomenon in greater detail, including the role of ART in mitigating HIV-related renal damage. Finally, these findings may encourage local stakeholders in adapting locally available evidence-based decisions and promote policymakers from other countries to review current guidelines or to strengthen further evidence from their own settings.

## Conclusion

In conclusion, our study provides important empirical evidence from real life experience that TDF did not result in a significant decline in renal function among PLHIV. Indeed, an unexpected trend of improved renal function was observed. This suggests that TDF can be administered safely in African settings where ART decisions are frequently made without pre-treatment renal assessments. While these findings are encouraging, they should be interpreted with caution due to the study’s significant attrition rate.

## Supplementary Information

Below is the link to the electronic supplementary material.

Supplementary Material 1

## Data Availability

The datasets used and/or analysed during the current study are available from the corresponding author on reasonable request.

## Abbreviations

ART: Antiretroviral therapy
BP: Blood pressure
CG: Cockcroft-Gault equation
CKD: Chronic kidney disease
CKD-EPI: Chronic Kidney Disease Epidemiology Collaboration equation
EFV: Efavirenz
eGFR: estimated glomerular filtration rate
INSTI: Integrase inhibitor
IQR: Interquartile range
KCH: Kamuzu Central Hospital
LighTen: Lighthouse Tenofovir cohort study
MAP: Mean arterial pressure
MDRD: Modification of Diet in Renal Disease equation
NCD: Non-communicable diseases
NNRTI: Non-nucleoside reverse transcriptase inhibitor
NRTI: Nucleoside reverse transcriptase inhibitor
PLHIV: People living with HIV
SD: Standard deviation
SSA: sub-Saharan Africa
TDF: Tenofovir disoproxil fumarate
WHO: World Health Organization
