# Supplementary material for: Renal function during tenofovir‑based antiretroviral therapy among people living with HIV in Lilongwe, Malawi: findings from the prospective LighTen cohort study
Source: BMC Infect Dis. 2026 Jan 28;26:416. doi: 10.1186/s12879-026-12675-2 (PMC12924523; doi:10.1186/s12879-026-12675-2)
Supplement: Supplementary file 1 — Supplementary Material 1 [file 12879_2026_12675_MOESM1_ESM.docx]

Supplementary Materials

**Renal function during tenofovir‑based antiretroviral therapy among people living with HIV in Lilongwe, Malawi: findings from the prospective LighTen Cohort Study**

Melani R. MAHANANI^1,2^, Florian NEUHANN^1,3^, Ethel RAMBIKI^4^, Angelina NHLEMA^4^, Hannock TWEYA^5^, Myo CHIT^1^, Jane CHIWOKO^4^, Thom CHAWEZA^4^, Claudia WALLRAUCH^4,6^, Tom HELLER^4,7^, Volker WINKLER^1^, Gerd FÄTKENHEUER^8^, Hans-Michael STEFFEN^9,10,11^

^1^Heidelberg Institute of Global Health, Heidelberg University Hospital, Heidelberg, Germany.

^2^Centre for Preventive Medicine and Digital Health, Division of Prevention of Cardiovascular and Metabolic Conditions, Medical Faculty Mannheim, Heidelberg University, Mannheim, Germany.

^3^School of Medicine and Clinical Sciences, Levy Mwanawasa Medical University, Lusaka, Zambia.

^4^Lighthouse Clinic, Kamuzu Central Hospital, Lilongwe, Malawi.

^5^International Training and Education Center for Health (I-TECH), Lilongwe, Malawi.

^6^Institute of Infectious Diseases and Tropical Medicine, LMU University Hospital, LMU Munich, Germany.

^7^International Training and Education Centre for Health, University of Washington, Seattle, WA, USA.

^8^Division of Infectious Diseases, Department I of Internal Medicine, University of Cologne, Faculty of Medicine and University Hospital Cologne, Cologne, Germany

^9^Clinic for Gastroenterology and Hepatology, University of Cologne, Faculty of Medicine and University Hospital Cologne, Cologne, Germany.

^10^Hypertension Center, University of Cologne, Faculty of Medicine and University Hospital Cologne, Cologne, Germany.

^11^Department of Postgraduate Studies and Research, Chreso University, Lusaka, Zambia.


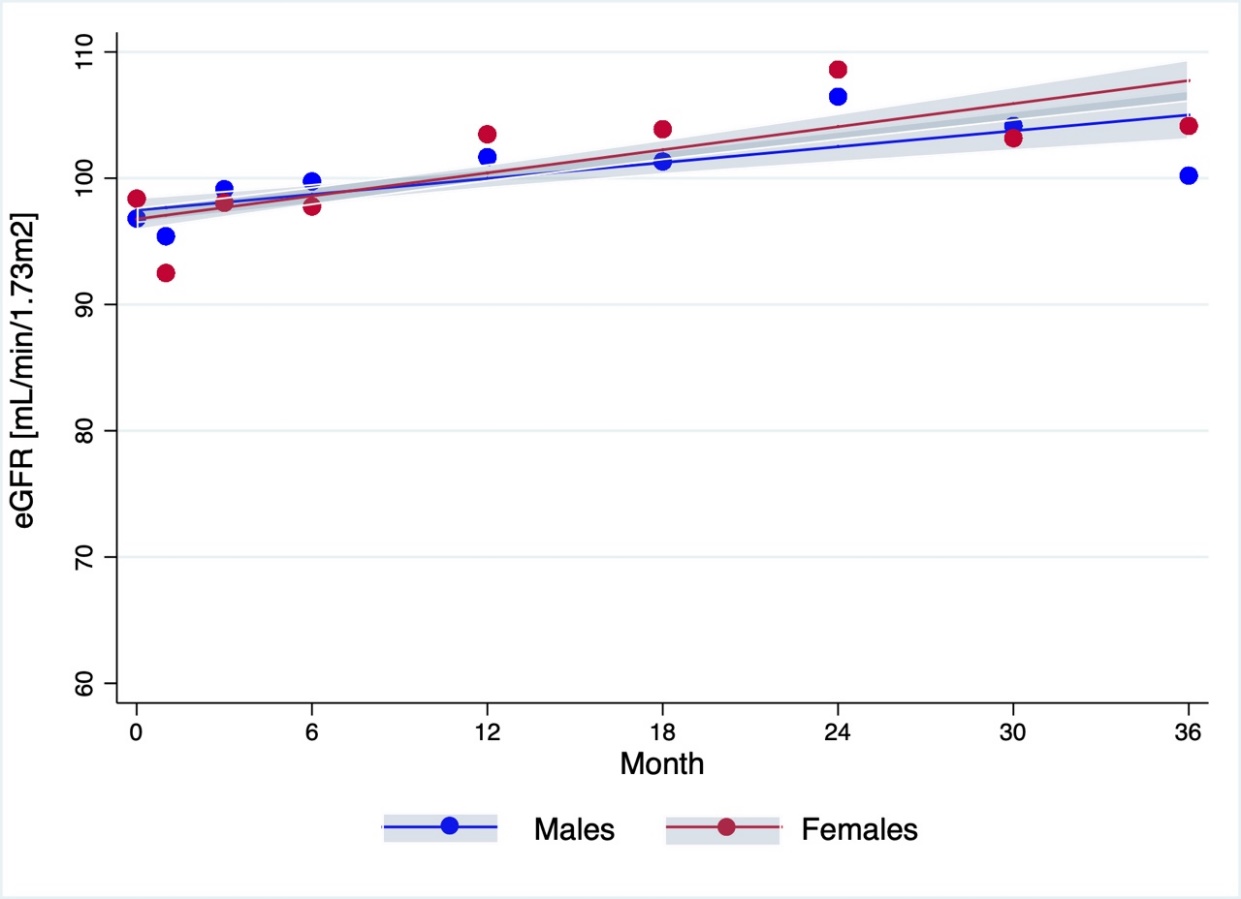


**Figure A1.** Mean eGFR over time from month 0 to month 36 along with modelled line and 95% confidence interval, separated by sex.


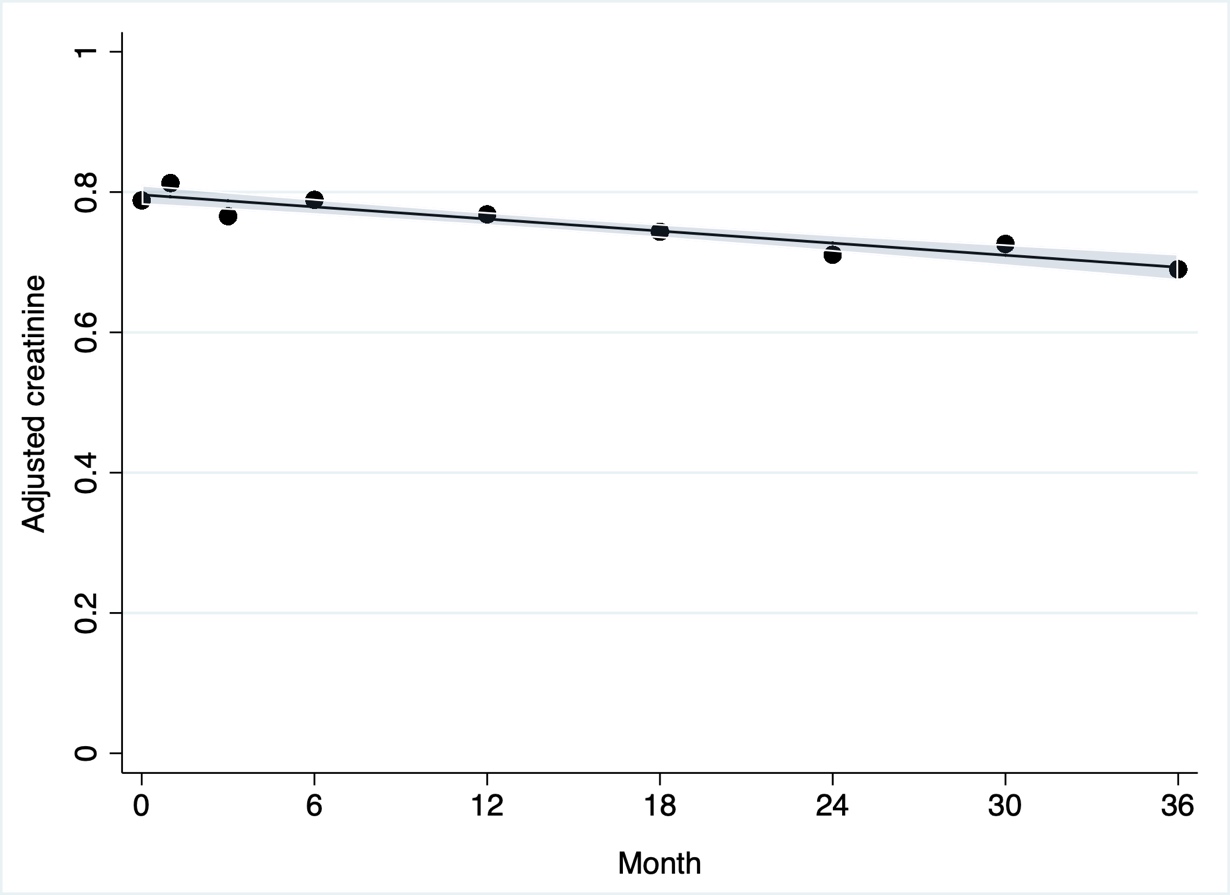


**Figure A2.** Changes of mean adjusted creatinine over time from month 0 to month 36 along with modelled line and 95% confidence interval.

**Table A1.** A cohort comparison of PLHIV who enrolled vs those who did not enrol in the period 2014-2016.

| Baseline Characteristics | | Total  n=2503 | | Enrolled  n= 1433 (57%) | | Not enrolled  n=1070 (43%) | | p-value^b^ |
| --- | --- | --- | --- | --- | --- | --- | --- | --- |
| Sex | |  |  |  |  |  |  | 0.453 |
| Male | | 1097 | (44%) | 618 | (43%) | 479 | (45%) |  |
| Female | | 1406 | (56%) | 815 | (57%) | 591 | (55%) |  |
| Age at ART start (years) | |  |  |  |  |  |  | 0.001 |
| 18-35 | | 1340 | (54%) | 727 | (51%) | 613 | (57%) |  |
| 36-55 | | 1057 | (42%) | 649 | (45%) | 408 | (38%) |  |
| 56+ | | 106 | (4%) | 57 | (4%) | 49 | (5%) |  |
| Mean±SD | | 35.9±9.6 | | 36.5±9.3 | | 35.1±9.9 | |  |
| BMI^a^(kg/m^2^) | |  |  |  |  |  |  | 0.001 |
| <18.5 | | 178 | (7%) | 82 | (6%) | 96 | (9%) |  |
| 18.5-24.9 | | 15191 | (61%) | 868 | (61%) | 651 | (61%) |  |
| 25-29.9 | | 545 | (22%) | 317 | (33%) | 228 | (22%) |  |
| >30 | | 254 | (10%) | 166 | (12%) | 88 | (8%) |  |
| Mean±SD | | 23.9±4.7 | | 24.2±4.9 | | 23.5±4.5 | |  |
| WHO stage at ART start | |  |  |  |  |  |  | 0.010 |
| WHO stage 1 | | 1184 | (47%) | 658 | (46%) | 526 | (49%) |  |
| WHO stage 2 | | 402 | (16%) | 223 | (16%) | 179 | (17%) |  |
| WHO stage 3 | | 718 | (29%) | 448 | (31%) | 270 | (25%) |  |
| WHO stage 4 | | 199 | (8%) | 104 | (7%) | 95 | (9%) |  |
| ART outcome^a^ | |  |  |  |  |  |  | 0.001 |
| Alive | | 1286 | (51%) | 803 | (56%) | 483 | (45%) |  |
| Dead | | 91 | (4%) | 54 | (4%) | 37 | (3%) |  |
| Defaulted | | 745 | (30%) | 398 | (28%) | 357 | (33%) |  |
| Stop ART | | 14 | (1%) | 9 | (1%) | 5 | (1%) |  |
| Transfer Out | | 356 | (14%) | 168 | (12%) | 188 | (18%) |  |
| Follow-up on ART (months) |  |  |  |  |  |  | 0.001 |  |
| <6 | 571 | (23%) | 271 | (19%) | 300 | (28%) |  |  |
| 6-12 | 204 | (8%) | 116 | (8%) | 88 | (8%) |  |  |
| 13-24 | 194 | (8%) | 113 | (8%) | 81 | (8%) |  |  |
| 25+ | 1528 | (61%) | 930 | (65%) | 598 | (56%) |  |  |
| Mean±SD | 31.0±21.6 | | 32.1±20.4 | | 29.6±23.4 | |  |  |

^a^Missing records: BMI=7; Reason for ART=59; ART outcome=1; Period on ART=36

^b^Chi2 test, α=0.05

**Table A2.** Multivariable linear regression for changes in adjusted creatinine over time from month 0 to month 36.

| **Variables** |  | **Model 1** | | **Model 2** | | **Model 3** | | **Model 4** | | **Model 5** | |
| --- | --- | --- | --- | --- | --- | --- | --- | --- | --- | --- | --- |
|  |  | n=443 | | n=443 | | n=368 | | n=368 | | n=352 | |
|  |  | **ß** | **p-value** | **ß** | **p-value** | **ß** | **p-value** | **ß** | **p-value** | **ß** | **p-value** |
| Adjusted creatinine at baseline |  | -0.915 | <0.001 | -0.917 | <0.001 | -0.928 | <0.001 | -0.929 | <0.001 | -0.928 | <0.001 |
| MAP at baseline |  | <0.001 | 0.128 | <0.001 | 0.103 | <0.001 | 0.238 | <0.001 | 0.240 | <0.001 | 0.134 |
| WHO HIV stage | 1 or 2 | - | - | Ref. | 0.143 | Ref. | 0.181 | Ref. | 0.174 | Ref. | 0.229 |
|  | 3 or 4 | - | - | 0.025 |  | 0.027 |  | 0.027 |  | 0.024 |  |
| Dipstick proteinuria | 0 | - | - | - | - | Ref. | 0.694 | Ref. | 0.682 | Ref. | 0.553 |
|  | 1+^a^ | - | - | - |  | 0.015 |  | 0.016 |  | 0.023 |  |
| CD4 count at baseline | ≥200 | - | - | - | - | - | - | Ref. | 0.696 | Ref. | 0.378 |
|  | <200 | - | - | - | - | - | - | -0.020 |  | -0.046 |  |
| log (viral load) at baseline | <4 | - | - | - | - | - | - | - | - | Ref, | 0.453 |
|  | 4 to <5 | - | - | - | - | - | - | - | - | 0.027 |  |
|  | 5 to <6 | - | - | - | - | - | - | - | - | -0.057 |  |
|  | ≥6 | - | - | - | - | - | - | - | - | -0.071 |  |
| Constant |  | 0.623 | <0.001 | 0.542 | <0.001 | 0.563 | <0.001 | 0.564 | <0.001 | 0.614 | <0.001 |

^a^Dipstick proteinuria (1+ or more).
